# Falls Assessment Clinical Trial (FACT): design, interventions, recruitment strategies and participant characteristics

**DOI:** 10.1186/1471-2458-7-185

**Published:** 2007-07-29

**Authors:** C Raina Elley, M Clare Robertson, Ngaire M Kerse, Sue Garrett, Eileen McKinlay, Beverley Lawton, Helen Moriarty, A John Campbell

**Affiliations:** 1 Department of General Practice and Primary Health Care, School of Population Health, University of Auckland, New Zealand; 2 Department of Medical and Surgical Sciences, Dunedin School of Medicine, University of Otago, New Zealand; 3 Department of Primary Healthcare and General Practice, School of Medicine and Health Sciences, University of Otago, Wellington, New Zealand

## Abstract

**Background:**

Guidelines recommend multifactorial intervention programmes to prevent falls in older adults but there are few randomised controlled trials in a real life health care setting. We describe the rationale, intervention, study design, recruitment strategies and baseline characteristics of participants in a randomised controlled trial of a multifactorial falls prevention programme in primary health care.

**Methods:**

Participants are patients from 19 primary care practices in Hutt Valley, New Zealand aged 75 years and over who have fallen in the past year and live independently. Two recruitment strategies were used – waiting room screening and practice mail-out. Intervention participants receive a community based nurse assessment of falls and fracture risk factors, home hazards, referral to appropriate community interventions, and strength and balance exercise programme. Control participants receive usual care and social visits. Outcome measures include number of falls and injuries over 12 months, balance, strength, falls efficacy, activities of daily living, quality of life, and physical activity levels.

**Results:**

312 participants were recruited (69% women). Of those who had fallen, 58% of people screened in the practice waiting rooms and 40% when screened by practice letter were willing to participate. Characteristics of participants recruited using the two methods are similar (p > 0.05). Mean age of all participants was 81 years (SD 5). On average participants have 7 medical conditions, take 5.5 medications (29% on psychotropics) with a median of 2 falls (interquartile range 1, 3) in the previous year.

**Conclusion:**

The two recruitment strategies and the community based intervention delivery were feasible and successful, identifying a high risk group with multiple falls. Recruitment in the waiting room gave higher response rates but was less efficient than practice mail-out. Testing the effectiveness of an evidence based intervention in a 'real life' setting is important.

**Trial registration:**

Australian Clinical Trials Register ID 12605000054617.

## Background

Falls are a major cause of morbidity among older adults, with at least 30% of community-dwelling adults over 65 years of age falling each year [1,2]. The health and economic burden of falls is large, particularly related to falls resulting in serious injuries such as hip fractures [3,4]. Adults over 75 years are at the highest risk of falling. Primary health care is a key setting to identify older people at risk of falling.

Multifactorial interventions for falls prevention are particularly effective and are recommended for the prevention of community based falls among older people [5]. Interventions aimed at those at highest risk, particularly those who have fallen previously demonstrate the greatest benefit [6]. Those in the oldest age groups also benefit most, particularly from exercise interventions [7]. Successful trials of exercise programmes focussing on muscle strengthening, balance and walking have been delivered at home [8-11], in a retirement village [12] and in the community [13].

The Prevention of Falls in the Elderly Trial (PROFET) found that a structured interdisciplinary assessment for older people presenting to a hospital emergency department in the United Kingdom after a fall reduced subsequent falls and hospitalisations (odds ratios 0.39 and 0.61, respectively) [14]. The intervention involved detailed medical assessment by a geriatrician with appropriate referral, as well as home based occupational therapy review assessing for environmental hazards with education and advice.

Other single factor interventions that have produced reductions in fall rates include withdrawal of psychotropic medication [15], home hazard assessment [16,17], vitamin D supplementation (meta-analysis) [18] and group-led exercise interventions [12,13]. However, there is recent evidence that while oral calcium and vitamin D supplementation in healthy post-menopausal women improve hip bone density, they do not reduce risk of hip fracture and they increase risk of kidney stones [19]. There is inconclusive evidence that nutritional or behavioural interventions reduce falls [6,20]. Expedited surgery for removal of a first cataract significantly reduced falls [21] while removal of a second cataract did not [22]. A very recent trial showed that comprehensive vision assessment and treatment was associated with an increased risk in falls, although these results were not available at commencement of the current trial [23].

Although there is extensive evidence that some interventions in certain settings among particular populations are successful, research about translating this evidence into practice is needed. It is not clear whether interventions such as those used in the PROFET trial would reduce falls in older people identified in primary health care who had not yet had a fall-related injury. Some home exercise programmes for falls prevention have drawn participants from primary health care, but few studies have examined the use of comprehensive assessment and management of falls risk in this setting [6].

Trials large enough to detect a clinically significant effect are recommended. Interventions and outcome measures could be better described and standardised using a recognised taxonomy, which would allow reproduction and pooling of results. These standards and a taxonomy for interventions are being addressed by the Prevention of Falls Network Europe (ProFaNE) initiative [24]. Economic evaluations are recommended as part of evaluations of interventions. More trials have been recommended to assess the impact on injuries of evidence-based falls prevention interventions [25]. Therefore, standard definitions of falls and outcome measures and analyses have been proposed to allow pooling of results.

Recruitment of participants from multiple primary health care settings into a lifestyle programme or trial can have practical problems [26]. There are also risks of selection bias and low recruitment rates if recruitment relies on family physicians identifying potential participants [27,28]. Other primary health care trials have successfully used placement of research nurses in waiting rooms to ensure systematic and consecutive screening of potential participants, and prompt enrolment and measurement of baseline measures to minimise the selection bias, improve rate of recruitment and reduce the burden on the family physician or practice [29]. Therefore, this method was proposed for the Falls Assessment Clinical Trial (FACT) recruitment strategy. However, recruitment was time-consuming using this method, so a second recruitment strategy was added, using a systematic mail-out to all those in the study age group of each practice. The use of patients' registers to recruit participants has been shown to be marginally more efficient and cost-effective than recruitment during primary health care visits in a previous study of frail older adults [30]. The effect on participation rates, participant characteristics and generalisability of each recruitment strategy has been investigated in this paper.

This paper also describes the design, intervention and characteristics of study participants at baseline in the trial. FACT is a unique study in that it is a community based trial that tests the implementation of an evidence-based falls prevention intervention based in primary health care. In particular, the intervention combines falls-related medical assessment, home hazards assessments, bone health assessment, an exercise programme and a referral pathway coordinated by a community based nurse working with several primary health care practices. FACT uses standardised definitions and outcome measures recommended by ProFaNE to allow pooling of results with other trials. The FACT study design will also incorporate an economic evaluation if the intervention is found to be effective in reducing falls.

## Methods

### Aims

FACT aims to assess the effectiveness and cost-effectiveness of a primary health care, individually tailored, multifactorial falls prevention intervention in reducing falls and improving the quality of life and functioning among older people at risk of falling.

### Design

The study uses an individual randomised controlled trial design with one year of follow-up and a prospective cost-effectiveness evaluation. The cost-effectiveness evaluation takes a health funder and societal perspective. The Wellington Ethics Committee approved the study in September 2004 (ID number: 04/08/064).

### Study population

Adults aged 75 years and older (over 55 years for Maori and Pacific people) who had fallen in the last 12 months were identified within primary health care by two methods: waiting room recruitment in the first seven practices and postal invitations using the practice registers in all practices except the initial practice. Exclusion criteria include being unable to comprehend study information and consent processes, unstable or progressive medical condition, severe physical disability, or dementia (less than 7 on the Abbreviated Mental Test Score) [31].

### Recruitment

**Figure 1:**
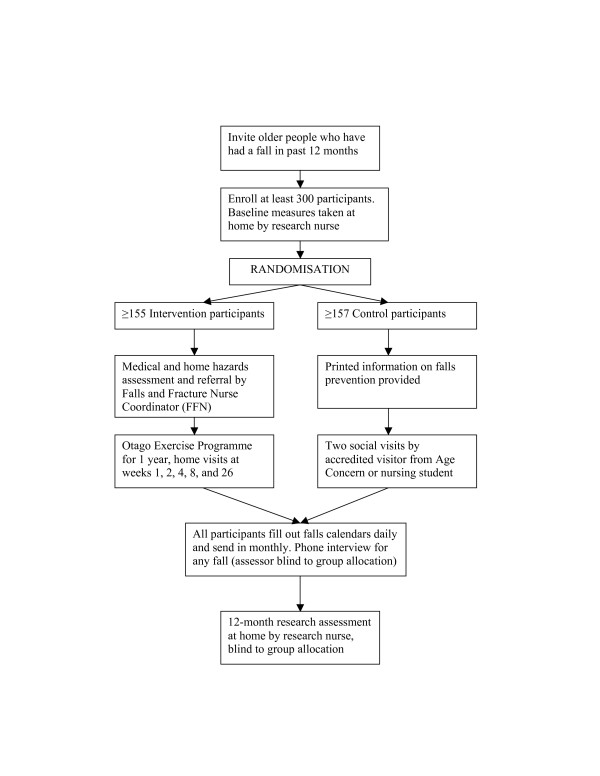
Participant and intervention flow through Falls Assessment Clinical Trial.

Rolling recruitment of participants from 19 practices in the Hutt Valley region in New Zealand was undertaken from March 2005 to January 2006 (Figure 1). Adults in the eligible age group were screened using a simple question asking if they had a fall or trip in the last 12 months on a form which also described the study briefly, handed to them by the receptionist as they entered the primary health care practice, or by mail-out to all those in the age group from each practice's patient register. If those who had fallen were interested in knowing more about the study from a research nurse, they provided their name and contact phone number and handed the form back to the receptionist or sent the form to researchers by post-paid envelope. The research nurse contacted those interested to provide more information about the study, confirm eligibility (e.g. that a fall meeting the study definition had occurred), and arrange a home visit if appropriate to conduct informed consent and baseline assessment.

### Randomisation and blinding

An independent researcher at a distant site carried out computer randomisation of participants, emailing allocation of randomisation of each individual after baseline assessment. The research nurses who undertake outcome measures at each time point remain blind to allocation to minimise measurement bias, although blinding is difficult where the participant is aware of which group they are in and home alterations may be evident at follow-up in the homes of some intervention participants.

### Outcome measures

Falls are defined as "an unexpected event in which the participants come to rest on the ground, floor, or other lower level" [25] and are the primary outcome measure. Falls are recorded by participants using postcard calendars, completed daily and posted monthly to the research team. If a fall is indicated on the calendar, a follow-up telephone interview establishes the circumstances and consequences of the fall from the participant, including injury and hospital admission. In a few cases, reports were confirmed from hospital records. Injuries are classified as serious (resulting in a fracture, hospital admission or sutures) or moderate (resulting in bruising, sprains, cuts, abrasions, seeking medical attention or a decrease in physical function for a period greater than or equal to three days) [7].

Secondary outcomes measured at baseline and 12 months are collected by the research nurse and include self-efficacy (modified fear of falling scale [32]), quality of life (SF-36 [33-36]), muscle strength and balance (timed up and go test, 30-second chair stand test [37], FICSIT 4-test balance scale [38] and 7.5 cm block step test [39]), activities of daily living (Nottingham extended activities of daily living profile [40,41]) and level of physical activity (Auckland Heart Study (AHS) physical activity questionnaire [42]). The timed up and go test measures the time taken to stand from a chair, walk three metres and return to the chair. The 30-second chair stand test measures the number of times the participant is able to stand and return to a seated position in a chair in 30 seconds [37]. The step test measures the number of times the person is able to step one foot fully onto and then off a 7.5 cm block in 15 seconds, repeated for each leg [39]. The average of the two legs is taken for each individual. The FICSIT (Frailty and Injuries: Cooperative Studies of Intervention Techniques) 4-test balance scale requires the participant to adopt four standing balance poses [38].

Other demographic and health variables were collected. Number of medical conditions was collected by self-report from a list of 27 common medical conditions including conditions such as arthritis, depression, diabetes, ischaemic heart disease and stroke.

### Cost variables

Unit costs and resource use of the components of the intervention, and costs associated with each fall to the participant and to the health funder, are being recorded over the 12-month duration of the study. If the intervention is found to be effective, then cost-effectiveness ratios will be calculated for incremental cost per fall averted [43].

### Intervention group

The intervention incorporates aspects from the successful PROFET trial [14], Tinetti's multifactorial intervention trial [44], and the individually tailored Otago Exercise Programme (Table 1) [45]. Intervention participants receive a falls risk assessment by a community based 'Falls and Fracture' nurse coordinator in their own home usually within one month of enrolment. The 'Falls and Fracture' nurse coordinator has gerontological expertise, and was trained in falls prevention. In brief, the intervention includes the following:

**Table 1:** Intervention protocol for the Falls Assessment Clinical Trial

**Falls prevention health assessment**	**Management**	**Falls & Fracture Nurse action**	**Referral**
*Circumstances of previous fall*	Changes in environment and activity to reduce risk of further falls	Directs further assessment and management. Address any immediate safety issues	Referral according to categories below

*Medication review:* High risk medications: benzodiazepines, other sleeping medications, neuroleptics, antidepressants, anti-convulsants [15]	Review and reduction of medications [6, 15]	Request family physician review of medications according to protocol	Family physician or geriatrician

*Vision:* Acuity < 20/60; decreased depth perception, contrast sensitivity (Melbourne edge test) [21, 50]	Ample lighting without glare; avoidance of multifocal glasses while walking	Arrange correction of lighting, highlight potential hazard edges	Optometrist, ophthalmologist (e.g. cataracts), family physician, geriatrician; if visual acuity 6/24 or worse, offer referral to Royal NZ Foundation of the Blind

*Postural blood pressure *(after > 5 mins in a supine position, immediately after standing, and 2 minutes after standing): Standing systolic blood pressure < 100 mmHg *or *= 20 mmHg postural drop if systolic blood pressure < 130 mmHg *or *with symptoms (immediate or > 2 mins)	Diagnosis and treatment of underlying cause	Adequate hydration, compensatory strategies (e.g. elevation of head of bed, rising slowly, dorsiflexion exercises), pressure stockings	Family physician or geriatrician: diagnosis and treatment of underlying cause, review and reduction of medications, or pharmacological therapy for postural hypotension

*Balance and gait:* Patient's report or observation of unsteadiness. Impairment on brief assessment (timed up and go test [48, 51, 52], 4-test balance scale [38])	Diagnosis and treatment of underlying cause	Coordinate Otago Exercise Programme (see below) or referral as appropriate	Family physician or geriatrician review Physiotherapist: assistance devices, supervised gait and progressive balance training if specific neurological problem or unable to do Otago Exercise Programme

*Targeted neurologic examination:* Impaired proprioception [53]; decreased muscle strength (chair stand test [37])	Diagnosis and treatment of underlying cause	Increase proprioceptive input (assistance device, appropriate footwear), caretaker's awareness of cognitive deficits	Family physician or geriatrician: review medications that impede cognition Physiotherapist: supervised gait, balance and strength training

*Targeted musculoskeletal examination*: Legs (joints and range of motion) and examination of feet to identify problems interfering with function	Diagnosis and treatment of underlying cause	Offer Otago Exercise Programme (see below) or referral as appropriate	Physiotherapist: supervised strength, range-of-motion, gait and balance training, assistance devices, appropriate footwear; Podiatrist or chiropodist: assist with feet Family physician address impairments (e.g. osteoarthritis)

*Targeted cardiovascular examination*: Syncope or arrhythmia [54, 55]			Family physician or geriatrician for ECG ± cardiologist referral, carotid-sinus massage (in case of syncope) [56]

*Continence/overactive bladder:* Particularly if related to circumstances of previous fall [57]		Nightlights, bladder retraining	Continence service/nurse for assessment, bladder retraining; family physician or geriatrician for medical management, exclusion of other pathology

**Home hazards assessment**			

Hazard identified according to protocol [14]	Changes in environment to reduce risk of further falls	Identify and modify minor home hazards (e.g. remove loose rugs, use nightlights)	Occupational therapist assessment for major hazards (e.g. bath/toilet grab rails) [17]

**Bone health assessment**			

Osteoporosis risk from osteoporosis screen questionnaire	Consider calcium and vitamin D supplementation if not receiving		Refer to family physician for appropriate management with suggestion of vitamin D and calcium supplementation with guidelines [18, 46]

Previous fragility fracture [58]	Consideration for appropriate management (including bisphosphonates) [58]	Organise vouchers and referral	Referral for voucher for DEXA scan and review by family physician for application for bisphosphonates [47]

**Otago Exercise Programme **[10, 45]			

All participants	Increase muscle strength and balance	Offer delivery of Otago Exercise Programme	Otago Exercise Programme delivered by accredited physiotherapist or nurse [45]

Unable to commence the Otago Exercise Programme or chronic neurological problem (e.g. existing CVA, Parkinson's disease), timed up and go test > 30 seconds, *or *cognitive impairment	Increase muscle strength and balance	Referral	Referral to physiotherapist for individualised rehabilitation programme

NZ denotes New Zealand.

• *Health assessment*: history of circumstances of the fall, medications, previous cardiovascular or neurological illness, continence, vision, postural blood pressure, balance and gait [37,38], cardiovascular screen (syncope, arrhythmia).

• *Home hazards assessment*: an audit for environmental safety [14,17].

• *Bone health assessment*: a brief osteoporosis risk screen, recommendation for vitamin D and calcium supplementation [46]. DEXA scan and bisphosphonates where indicated [47].

• *The Otago Exercise Programme *[45].

The nurse provides appropriate advice, education and coordinated medical referral to the family physician, geriatrician, optometrist, physiotherapist, occupational therapist or other professional if indicated, according to the assessment algorithm. Where indicated, the family physician undertakes a more comprehensive medical assessment, medication review and further referral or intervention where appropriate. A month-long pilot of the intervention involving 10 participants from one practice had established the feasibility and operational logistics of the falls and fracture nurse assessment and referral systems prior to the main trial.

The nurse coordinator organises the delivery of the Otago Exercise Programme by a trained health practitioner or physiotherapist for one year. The exercise programme involves home visits at weeks 1, 2, 4, 8 and after six months to administer the individually tailored strength and balance retraining programme. If the nurse considers the participant would not be able to undertake, or receive benefit from this exercise programme (e.g. timed up and go score of greater than 30 or marked neurological impairment) then she can refer the participant to a community physiotherapist who tailors an alternative exercise programme more appropriate for the participant.

### Control group

In addition to usual care, participants in the control group are offered at least two social visits from an accredited visitor such as a nursing student, within one month of enrolment, to control for the effect of social contact by the falls and fracture nurse coordinator and exercise initiator in the intervention group. Control participants also receive a pamphlet produced by the New Zealand Accident Compensation Corporation about prevention of falls in older adults.

### Sample size

On the basis of previous falls rates and attrition rates during a similar falls prevention programme we predicted the proportion of the control group and intervention group who will fall during a one year period to be 52% and 32% respectively [14]. To detect this as statistically significant (alpha = 0.05, power = 0.80), 105 participants were required in each group. If an attrition rate of 30% over the 12 months were assumed then a sample of 300 would be required (150 in each of the control and intervention groups).

### Analysis

Descriptive statistics are presented and intervention and control groups checked for balance in demographic, health and outcome measures. Recruitment rates and characteristics of participants recruited from the two recruitment strategies are compared.

For the main outcome results at the completion of the study, an intention to treat analysis will be undertaken. The rate of falls for individuals in the two groups will be compared using negative binomial regression models in STATA 9.1. Linear and logistic regression will be used to compare changes in the intermediate measures from baseline to 12 months. Differences in these outcomes between the intervention and control groups will be tested using the regression models, adjusting for baseline values.

## Results

### Recruitment rates

**Figure 2:**
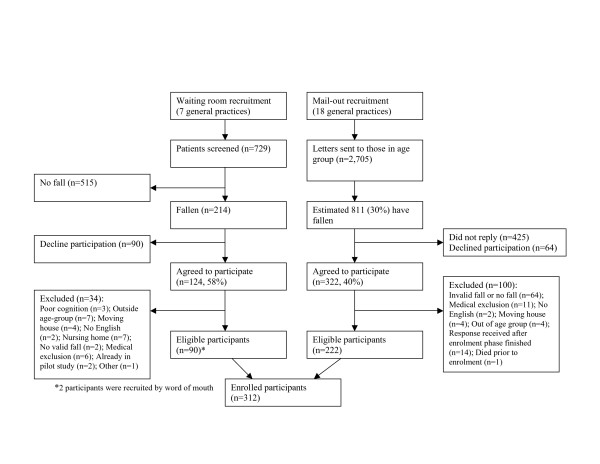
Recruitment using two recruitment strategies into the Falls Assessment Clinical Trial.

A total of 312 participants were recruited using two methods of recruitment from 19 primary health care practices. As a proportion of those screened, the recruitment rate for the waiting room method (12.3% (90 of 729)) was higher than for the postal method (8.2% (222 of 2705)) (p < 0.001). Figure 2 shows the recruitment of participants into the trial using the two methods. Screening in the practice waiting room revealed that 29% (214 of 729) of those screened had had a fall in the previous 12 months. Of those who had fallen, 58% (n = 124) agreed to participate, of whom 73% (n = 90) were eligible. Using the postal method of recruitment, if a fall rate of 30% is assumed (811 of 2705), then 40% agreed to participate (322 of 811). To test this assumption, if fall rates of 20% or 40% were assumed, then 59% (322 of 541) or 30% (322 of 1082), respectively, agreed to participate. Of those who agreed to participate 69% fulfilled eligibility criteria (n = 222).

### Characteristics of participants using different recruitment strategies

There was no statistically significant difference in participant demographic, clinical or functional measures between the two different recruitment strategies. There was a non-significant trend for those recruited by the mail-out compared with waiting room recruitment to be slightly younger (mean of 80.6 and 81.2 years, respectively (p = 0.4)), lower weight (body mass index 27 and 28 (p = 0.3)), with fewer medical conditions (6.9 and 7.5 (p = 0.1)) and fewer medications (5.4 and 5.8 (p = 0.3)), slightly better function on tests of strength and balance (timed up and go 14.5 and 16.6 seconds (p = 0.1); step test 8.5 and 7.5 steps (p = 0.07)) and a slightly higher proportion of women (71% and 63% (p = 0.1)). In addition, there was also a trend towards greater numbers of falls in the past 12 months compared with the waiting room recruitment group (3.4 and 2.7 (p = 0.2)).

### Baseline characteristics

In the total study sample, 31% (97 of 312) were men. Most participants (n = 303) were between 75 and 98 years of age. However, nine Maori or Pacific participants were between the ages of 60 and 75 years of age because of the different age eligibility criteria for these groups. In total, 276 (88%) participants identified as New Zealand European, 6 (2%) as Maori, 3 (1%) Pacific, one Indian, one Chinese and 29 (9%) identified as other European or other ethnicity. All participants had fallen in the last 12 months, eight (2.5%) had had a previous hip fracture, and 107 (34%) had had any previous fracture in the past. The total study population took an average of 5.5 medications with 29% taking psychotropic medications. Functional measures showed a limited level of function with an average timed up and go score of 15 seconds. The demographic and clinical characteristics by intervention and control groups were balanced at baseline (see Table 2).

**Table 2:** Demographic and clinical characteristics of all study participants

**Characteristic**	**Intervention **(n = 155)	**Control **(n = 157)	**Total **(n = 312)
Age, years	80.4 (4.8)	81.1 (5.3)	80.8 (5.0)
Female n (%)	105 (67%)	110 (70%)	215 (69%)
Number of falls in previous year Median [interquartile range]	2 [1,3]	2 [1,4]	2 [1,3]
Systolic blood pressure, mmHg	148.4 (24.2)	149.9 (21.7)	149.2 (22.9)
Diastolic blood pressure, mmHg	71.6 (11.9)	72.2 (11.4)	71.9 (11.6)
Body mass index, kg/m^2^	27.0 (6.0)	27.4 (4.7)	27.2 (5.4)
FICSIT 4-test balance score	3.5 (1.2)	3.5 (1.2)	3.5 (1.2)
Step test, number of steps	8.2 (4.5)	8.3 (4.5)	8.2 (4.5)
Timed up and go, seconds Median [interquartile range]	12 [10,16]	12 [10,16.5]	12 [10,16]
30 second chair stand, number of stands	8.5 (4.5)	8.2 (4.6)	8.3 (4.6)
Nottingham extended ADL score	18.4 (3.4)	18.1 (3.4)	18.3 (3.4)
Modified falls efficacy score	8.1 (1.8)	8.0 (1.9)	8.05 (1.83)
Number of medical conditions	6.9 (2.8)	7.2 (2.9)	7.0 (2.9)
Number of medications	5.3 (3.4)	5.6 (3.2)	5.5 (3.3)
Taking psychotropic medication(s) n (%)	48 (31%)	41 (26%)	89 (29%)
Previous cerebrovascular accident n (%)	28 (18%)	48 (31%)	76 (24%)
Previous fracture† n (%)	60 (39%)	47 (30%)	107 (34%)
Previous hip fracture† n (%)	2 (1%)	6 (4%)	8 (3%)
Leisure activity/walking, minutes/week Median [interquartile range]	120 [20, 250]	120 [13, 218]	120 [16, 240]

* Values are mean (SD) unless indicated otherwise. Median and interquartile range are used where results were not normally distributed

† Fracture at any age in the past.

FICSIT denotes Frailty and Injuries: Cooperative Studies of Intervention Techniques (range 0 – 5, higher scores indicate better balance). Step test: higher scores indicate better performance. Timed up and go: shorter times indicate better performance. 30 second chair stand: higher scores indicate better performance. Nottingham extended activities of daily living score: range 0 – 22, higher scores indicates better performance. Modified falls efficacy score: range 0 – 10, higher scores indicate increased confidence.

## Discussion

This paper describes the intervention, design, recruitment strategies and baseline results of the FACT falls prevention trial for older people with a history of falls from a range of primary health care practices. The intervention was multifactorial and the components were evidence-based. Baseline characteristics of intervention and control groups were balanced.

Tests of strength and balance confirmed that this population of older people screened in primary health care with a recent previous fall had relatively low physical function and generally high rates of morbidity and medications. For example, the mean timed up and go of this population was 15.1 seconds (median 12 seconds, interquartile range 10–16 seconds) compared with a mean of 8.5 seconds amongst healthy older people (mean age 75 years) measured by Podsiadlo and Richardson [48], and 9.1 seconds in a cohort of healthy older women (70 years and over) [39]. The mean step test of the FACT study population was 8.2 steps in 15 seconds compared with 17 steps in a study of healthy older people (mean age 73 years) that used the same step dimensions [32], and 16 steps among a healthy cohort of older women [39]. It is likely, therefore, that a strength and balance programme may benefit this population.

It has been shown that opportunistic recruitment, such as by newspaper advertisements, produce less diverse and representative patient populations than recruitment within the appropriate clinical context [49]. However, even if recruitment is undertaken within primary health care, relying on family physicians or practice staff to recruit participants can be problematic due to the time constraints of the staff, and can often produce low recruitment rates and selection bias [27,28]. Therefore, systematic screening and recruitment from primary health care waiting rooms and by mail-out screening from patient registers were undertaken in this study.

This study showed that systematic screening for previous falls and recruitment within the practice waiting room had higher rates of recruitment than mail-out screening from practice registers. Those identified from the practice waiting room were open to receiving the home based falls prevention programme, with 58% willing to participate compared with 40% using the mail-out strategy, although the latter figure may be imprecise as it assumed a fall rate of 30% in those in the study age group. When the characteristics of participants of the two recruitment strategies were compared there were no significant differences on general characteristics or outcome measures. Therefore, although the recruitment rate was lower using the mail-out method, the method was more time efficient than the consecutive screening and recruitment in the waiting rooms. Either method would be appropriate in future trials. The choice of method may depend on the prevalence of the condition.

## Conclusion

Although many of the intervention components have been found to be effective at reducing falls in older people, few studies have demonstrated the effectiveness and cost-effectiveness of a new health service combining falls-related medical assessment, home hazards assessment, bone health, exercise intervention and community based nurse coordination of referral and follow-up in a "real world" primary health care context. The multifactorial intervention approach is also testing for the interaction of interventions previously shown to be effective as single interventions in selected populations. Although this has been done previously (PROFET) [14], it has not been well investigated where implementation of a significant proportion of the interventions requires referral outside the research team, with limited control on uptake.

## Competing interests

The authors declare that they have no competing interests.

## Authors' contributions

CRE contributed to design, supervised all aspects of the study, contributed to analyses and interpretation of results, and drafted the manuscript. MCR contributed to the design, random sequence generation, analysis, interpretation and preparation of the manuscript. NK contributed to the design, interpretation of results and preparation of the manuscript. SG contributed to data collection and management, project management, analysis, interpretation of results and manuscript preparation. EM contributed to the study design, data collection supervision, interpretation of results and manuscript preparation. BL advised on study design, interpretation of results and manuscript preparation. HM contributed to the design of the study, intervention set-up, interpretation of results and manuscript preparation. AJC conceived of the intervention, contributed to the design, interpretation and manuscript preparation. All authors read and approved the final manuscript.

## Pre-publication history

The pre-publication history for this paper can be accessed here:


